# Modeling Sensor Reliability in Fault Diagnosis Based on Evidence Theory

**DOI:** 10.3390/s16010113

**Published:** 2016-01-18

**Authors:** Kaijuan Yuan, Fuyuan Xiao, Liguo Fei, Bingyi Kang, Yong Deng

**Affiliations:** 1 School of Computer and Information Science, Southwest University, Chongqing 400715, China; yuankaijuan@163.com (K.Y.); xiaofuyuan@swu.edu.cn (F.X.); feiliguoswu@163.com (L.F.); kangbingyiswu@163.com (B.K.); 2 School of Electronic and Information, Northwestern Polytechnical University, Xi’an 710072, China; 3 Big Data Decision Institute, Jinan University, Tianhe, Guangzhou 510632, China; 4 Department of Civil & Environmental Engineering, School of Engineering, Vanderbilt University, Nashville, TN 37235, USA

**Keywords:** sensor data fusion, sensor reliability, Dempster–Shafer evidence theory, belief function, Deng entropy, fault diagnosis, evidential conflict

## Abstract

Sensor data fusion plays an important role in fault diagnosis. Dempster–Shafer (D-R) evidence theory is widely used in fault diagnosis, since it is efficient to combine evidence from different sensors. However, under the situation where the evidence highly conflicts, it may obtain a counterintuitive result. To address the issue, a new method is proposed in this paper. Not only the statistic sensor reliability, but also the dynamic sensor reliability are taken into consideration. The evidence distance function and the belief entropy are combined to obtain the dynamic reliability of each sensor report. A weighted averaging method is adopted to modify the conflict evidence by assigning different weights to evidence according to sensor reliability. The proposed method has better performance in conflict management and fault diagnosis due to the fact that the information volume of each sensor report is taken into consideration. An application in fault diagnosis based on sensor fusion is illustrated to show the efficiency of the proposed method. The results show that the proposed method improves the accuracy of fault diagnosis from 81.19% to 89.48% compared to the existing methods.

## 1. Introduction

With the development of sensor data fusion technology, it is playing a more and more important role in fault diagnosis. On account of the complexity of the target and the background, the data detected by a single sensor are insufficient and unreliable to make a decision. In addition, due to the impact of the surroundings, the information derived from the sensors may contain errors, which leads to an incorrect result in the fault diagnosis system. A multi-sensor system can partially overcome the above limitations and shortages by combining a group of sensors to detect information and make a decision by considering all of the information obtained from the detection system [1,2], which improves the reliability and accuracy of the fault diagnosis system effectually [3,4].

In practical applications, the information collected from the sensors is imprecise and uncertain. How to deal with the uncertain information effectively to make a reasonable decision or optimization has had great attention paid [5,6]. To address this issue, some theories focused on uncertainty modeling and data fusion have been introduced, such as evidence theory [7,8], fuzzy set theory [9,10,11,12], Bayesian networks [13] and D-numbers [14]. Dempster–Shafer evidence theory (D-S evidence theory) is an imprecise reasoning theory, which was first proposed by Dempster [15] and then developed by Shafer [16]. As the generalization of Bayes method, D-S evidence theory can deal with uncertain information without prior probability. When the uncertain information is represented by probability, D-S evidence theory can definitely degenerate to the probability theory. D-S evidence theory is useful in uncertainty modeling [17] and data fusion [18,19,20], which contributes to its wide application in the fields of uncertain information processing [21,22,23] and decision making [24,25,26]. Cai etal. introduced the Bayesian network and proposed to establish two layers, a fault layer and a fault symptom layer, to develop a fault diagnosis model and to perform data fusion [27]. It should be pointed out that D-numbers can model and fuse more uncertain information, which is also an efficient math tool to handle data uncertainty [14,28,29].

However, there may exist conflict among the data collected from different sensors. In addition, the error contained in the data can also lead to conflict [30]. It may come to a counterintuitive conclusion by using Dempster’s combination rule when faced with highly conflicting evidence [31]. How to handle the conflict is inevitable in fault diagnosis. There are two classes of solutions to address the issue. The first is to improve the combination rule method, while the other is to modify the data model [32]. Yager improved the combination rule by distributing the conflict factor to the universal set, which means knowing nothing [33]. Smets introduced a conjunctive combination rule [34,35]. Dubios and Prade put forward a disjunctive combination rule [36,37]. Some typical works to improve the data method are briefly introduced as follows. Murphy is in favor of modifying evidence instead of the combination rule; she proposed to average the belief function first and perform the data fusion next [38]. Deng etal. introduced a weighted averaging method [39], which is more reasonable compared to Murphy’s simple averaging [40,41]. Zhang etal. introduced the vector space to deal with the issue [42].

Fan and Zuo introduced a fuzzy membership function and an importance index to improve D-S evidence theory [43]. Three factors are taken into consideration: evidence sufficiency, evidence importance and conflict degree of evidence. Though this method improves the accuracy of fault diagnosis, it still has some problems. First, it introduces a judging process of the conflict degree of evidence, according to the different combination rule being adopted, which makes it much more complex to make a decision. Besides, Fan and Zuo’s method only considers evidence sufficiency and evidence importance, which can be regarded as the static property of sensors’ reliability, and ignores the dynamic property of sensors reliability reflected in the real-time detection process.

It is obvious that the sensor reliability plays a significant role in decision making and fault diagnosis [44]. Sensor reliability can quantify sensor performance and reflect the reasonability of sensor data. In general, sensor performance is measured in long-term practice. This kind of reliability is called static reliability, which mainly depends on technical factors of the sensor itself. However, in a dynamic situation, with the surrounding conditions changing with time, the sensors may perform with different reliability at different times. It is difficult to measure such changeable reliability with one parameter in practical applications. Therefore, dynamic reliability is adopted to reflect the variation of sensor reliability at different times. Note that the reliability of a dynamic system is different from dynamic reliability on account of the reliability of a dynamic system being composed of static reliability and dynamic reliability. It can be considered that the reliability of a dynamic system is from the macro perspective, while the dynamic reliability is from the micro perspective. Additionally, the dynamic reliability approximates the real-time reliability. Cai etal. proposed to evaluate such dynamic reliability on the basis of dynamic Bayesian networks [45,46,47]. Rogova and Nimier have made a complete survey of evaluating the sensor reliability [48] in information fusion, which can be summed up as three levels: sensor level, data level and symbol level. The first level is inherent in a sensor, while the second and the third levels are application oriented [44]. Based on all of the above, this paper proposes a new method to model the reliability at two levels: The first level is static reliability, and the second level is dynamic reliability. The static reliability mainly depends on the technical factors, such as manufacturing craft and noise due to different materials. It can be measured by comparing the detection value with the actual value in long-term practice and the experts’ assessment. The dynamic reliability is influenced by the properties of the target and the surroundings. It can be evaluated by comparing the consistency of the outputs with other sensors aimed at the same input. If one sensor’s outputs are in great consensus with others, it is considered to have great reliability. The new method distributes different weights to different sensor data according to the sensor reliability and adopts the weighted averaging method to combine different evidence. The new method considering both the static reliability and the dynamic reliability of a sensor is more reasonable to cope with conflicting evidence effectually.

The proposed method has the following advantages. First, it is a generalized version of our previous work [44]. Compared to the existing method, the dynamic property of sensor reliability is not only determined by the evidence distance function, but also by the information volume of the sensor itself. It is more reasonable, since the information volume is an important parameter of the sensor report and should be taken into consideration in sensor data fusion. Second, the proposed method improves the accuracy in fault diagnosis, since it is efficient at conflict management. It is useful to practical engineering, since the methodology of this paper can be easily extended to other multi-sensor systems.

The paper is organized as follow. Section 2 introduces the preliminaries of the D-S evidence theory [15,16] and Deng entropy. Fan and Zuo’s method is briefly described in Section 2. Section 3 presents the new method to modeling sensor reliability. A numerical example is illustrated in Section 4 to show the efficiency of the new method. Finally, this paper is concluded in Section 5.

## 2. Preliminaries

In this section, some preliminaries are briefly introduced below.

### 2.1. Dempster–Shafer Evidence Theory

Dempster–Shafer evidence theory (D-S evidence theory) is also called belief function theory [15,16].

Let Θ be a set of *n* mutually-exclusive and collectively-exhaustive events, which is called the frame of discernment. The elements in Θ represent all of the possible faults in the fault domain of the object. Θ, also known as the sample space, is defined as $\Theta =\{\theta_{1},\theta_{2},\ldots ,\theta_{n}\}$. The power set of Θ is denoted by $2^{\Theta }$, whose element is called a hypothesis or a proposition. On the basis of the above two concepts, the definition of the mass function can be described. A mass function, also called basic belief assignment (BBA), is a mapping mfrom $2^{\Theta }$ to $\left[0,1\right]$, which is given below:

(1) $$m:2^{\Theta }\to \left[0,1\right]$$

Satisfying:

(2) $$\begin{array}{c} m\left(\emptyset \right)=0\\ \sum_{A\subseteq \Theta }m\left(A\right)=1 \end{array}$$

The value m(A) represents the belief degree distributed to hypothesis *A*. Note that m(⌀)=0 means that there is no belief degree assigned to the empty set, which is required in the closed world. While in the open world [30], the criterion is not required, and m(⌀) can be bigger than zero. All subsets *A* of Θ that satisfying m(A)>0 are called focal elements.

Dempster’s combination rule, also called the orthogonal sum, is defined as follows:

(3) $$m\left(C\right)=m_{i}\left(X\right)⊕m_{i}\left(Y\right)=\left\{\begin{array}{cc} 0 & X\cap Y=\emptyset \\ \frac{\sum_{X\cap Y=C,X,Y\subseteq \Theta }m_{i}\left(X\right)\times m_{i}\left(Y\right)}{1-K} & X\cap Y\neq \emptyset \; \end{array}\right.$$

*K* is called the conflict factor between m(X) and m(Y), which is defined below:

(4) $$K=\sum_{X\cap Y=\emptyset ,\forall X,Y\subseteq \Theta }m_{i}\left(X\right)\times m_{i}\left(Y\right)$$

When there are more than two pieces of evidence, these can be combined in the following form:

(5) $$m=m_{1}⊕m_{2}⊕\cdots ⊕m_{n}=\left(\left(\left(m_{1}⊕m_{2}\right)⊕\cdots \right)⊕m_{n}\right)$$

### 2.2. Weighted Average Combination Method [39]

In Dempster’s combination rule [15], *K* is adopted to measure the dissimilarity degree between BBAs. However, it does not respect the metric axioms under the conditions of identityand triangle inequality [44]. Here is a numerical example to illustrate the case.

Example 1: Assume there are two pieces of evidence, $m_{1}$ and $m_{2}$, whose BBAs are given below:

$m_{1}\left(\left\{\omega_{1}\right\}\right)=0.2,m_{1}\left(\left\{\omega_{2}\right\}\right)=0.2,m_{1}\left(\left\{\omega_{3}\right\}\right)=0.2,m_{1}\left(\left\{\omega_{4}\right\}\right)=0.2,m_{1}\left(\left\{\omega_{5}\right\}\right)=0.2,$

$m_{2}\left(\left\{\omega_{1}\right\}\right)=0.2,m_{2}\left(\left\{\omega_{2}\right\}\right)=0.2,m_{2}\left(\left\{\omega_{3}\right\}\right)=0.2,m_{2}\left(\left\{\omega_{4}\right\}\right)=0.2,m_{2}\left(\left\{\omega_{5}\right\}\right)=0.2.$

Use Equation (4) directly, and the conflict factor between two pieces of evidence is:

K=0.2×(0.2+0.2+0.2+0.2)×5=0.8

It is obvious that the two pieces of evidence are completely the same. However, the conflict factor is not equal to zero, which is not reasonable. In order to address the issue, Liu proposed a novel approach to measure the degree of conflict, which combines the conflict factor and betting comments [49]. Though it is useful in conflict measurement, it is too complex to be calculated. Jousselme etal. introduce a distance to measure the dissimilarity between two pieces of evidence [50]. The evidence is expressed in the form of the vector space. The distance between two pieces of evidence $m_{1}\left(\cdot \right)$ and $m_{2}\left(\cdot \right)$ denotes $d_{BOE}\left(m_{1},m_{2}\right)$, which is defined as:

(6) $$d_{BOE}\left(m_{1},m_{2}\right)=\sqrt{\frac{1}{2}{\left({\vec{m}}_{1}-{\vec{m}}_{2}\right)}^{T}\underline{\underline{D}}\left({\vec{m}}_{1}-{\vec{m}}_{2}\right)}$$

${\vec{m}}_{1}$ and ${\vec{m}}_{2}$ are the vector form of evidence, respectively. $\underline{\underline{D}}$ is a matrix of $2^{\Theta }\times 2^{\Theta }$, and the elements of $\underline{\underline{D}}$ are defined as:

(7) $$\underline{\underline{D}}\left(s_{1},s_{2}\right)=\frac{\left|s_{1}\cap s_{2}\right|}{\left|s_{1}\cup s_{2}\right|}\begin{array}{ccc} & & s_{1},s_{2}\in 2^{\Theta } \end{array}$$

When there are multiple pieces of evidence, the distances of every two pieces of evidence can be expressed in the form of a distance matrix DM, which is given below:

(8) $$DM=\left[\begin{array}{cccc} 0 & d_{12} & \cdots & d_{1m}\\ d_{21} & 0 & \cdots & d_{2m}\\ \vdots & \vdots & \vdots & \vdots \\ d_{m1} & d_{m2} & \cdots & 0 \end{array}\right]$$

On account of the distance measuring the dissimilarity of evidence, the greater the distance of two pieces of evidence is, the less the two pieces of evidence support each other, the greater the conflict between these pieces of evidence is. Thus, the similarity measure $Sim_{ij}$ can be defined:

(9) $$Sim\left(m_{i},m_{j}\right)=1-d\left(m_{i},m_{j}\right)$$

Additionally, the similarity measure matrix (SMM) is shown as:

(10) $$SMM=\left[\begin{array}{cccc} 1 & S_{12} & \cdots & S_{1m}\\ S_{21} & 1 & \cdots & S_{2m}\\ \vdots & \vdots & \vdots & \vdots \\ S_{m1} & S_{m2} & \cdots & 1 \end{array}\right]$$

The support degree of each evidence is given as:

(11) $$Sup\left(m_{i}\right)=\sum_{j=1,j\neq i}^{m}Sim\left(m_{i},m_{j}\right)$$

After normalization, the credibility degree $Crd_{i}$ of evidence *i* is given below:

(12) $$Crd_{i}=\frac{Sup\left(m_{i}\right)}{maxSup\left(m_{i}\right)}\begin{array}{ccc} & & \left(i=1,2,\cdots ,k\right) \end{array}$$

The bigger the Crd is, the more the evidence is supported by others, the more reliable it is and the more important the role it will play in the final fusion result.

There is no denying that D-S evidence theory [15] is effective in uncertainty modeling and data fusion. However, it may reach a counterintuitive conclusion when dealing with highly conflicting evidence. Zadeh has proposed such a numerical example [31]:

Example 4: Assume there are two pieces of evidence $m_{1}$ and $m_{2}$. The BBAs supported by such evidence are:

$m_{1}\left(\left\{F_{1}\right\}\right)=0.9,m_{1}\left(\left\{F_{3}\right\}\right)=0.1,$

$m_{2}\left(\left\{F_{2}\right\}\right)=0.9,m_{2}\left(\left\{F_{3}\right\}\right)=0.1.$

Use Equation (3), and the BBA of hypothesis $F_{3}$ is calculated as:

$m\left(\left\{F_{3}\right\}\right)=\frac{0.1\times 0.1}{1-0.9\times 0.1-0.1\times 0.9-0.9\times 0.9}=1$

The fusion result distributes total belief to $F_{3}$, while the two initial pieces of evidence do not support evidence $F_{3}$ well. Obviously, the final result deviates from reality, which may lead to the wrong decision. To handle this issue, Murphy introduced a simple averaging method to modify the BBAs [38]. Deng etal. proposed to apply the weighted averaging method, which is more reasonable [39]. In the weighted averaging method, different evidence plays different important roles in the final combination result according to the weights. If evidence has a big weight, it will have a great effect in the decision making; while if evidence is assigned a small weight, it will have little influence in the final fusion result. Assume there are *n* pieces of evidence; the weighted averaging method is summarized as:

(13) $$\begin{array}{c} m\left(A\right)=\sum_{i=1}^{n}w_{i}m_{i}\left(A\right)\\ \sum_{i=1}^{n}w_{i}=1 \end{array}$$

In fault diagnosis, the weights are given according to the efficiency of the evidence. The more reliable and accurate the evidence is, the higher the weight is. On the contrary, the less reliable and accurate the evidence is, the lower the weight distribution. In this paper, the weights are given based on the reliability of the sensors. The higher credibility a sensor has, the greater effect it will have on the final fusion and decision making.

### 2.3. Deng Entropy

Deng entropy is the generalization of Shannon entropy, which was first proposed by Deng [51]. It is an efficient way to measure uncertainty, not only under the situation where the uncertainty is represented by a probability distribution, but also the situation where the uncertainty is represented by BBA. Thanks to this advantage, Deng entropy is widely applied in D-S evidence theory. When the uncertainty is expressed in the form of a probability distribution, Deng entropy definitely degenerates to Shannon entropy. The related concepts are given below.

Let $A_{i}$ be a proposition of BBA *m*; the cardinality of the set $A_{i}$ is denoted by $\left|A_{i}\right|$. Deng entropy $E_{d}$ of set $A_{i}$ is defined as:

(14) $$E_{d}=-\sum_{i}m\left(A_{i}\right)\log\frac{m\left(A_{i}\right)}{2^{\left|A_{i}\right|}-1}$$

When the belief value is only assigned to a single element, Deng entropy can definitely degenerate to Shannon entropy, namely:

(15) $$E_{d}=-\sum_{i}m\left(A_{i}\right)\log\frac{m\left(A_{i}\right)}{2^{\left|A_{i}\right|}-1}=-\sum_{i}m\left(A_{i}\right)\log m\left(A_{i}\right)$$

For more detailed information, please refer to [51].

### 2.4. Fan and Zuo’s Method

Fan and Zuo proposed to improve the evidence by evidence sufficiency, evidence importance and the conflict among evidence [43]. In practical applications, the data obtained from a sensor may contain uncertainty and errors. Fan and Zuo introduced the fuzzy relationship function to measure evidence sufficiency, which denotes *μ*. Besides, not al of the pieces of evidence are of the same importance. Fan and Zuo introduced the evidence weight to represent evidence importance, which denotes *v*. The modification of BBAs considering both evidence sufficiency and evidence importance is given below:

(16) $$m_{i,•}\left(A\right)=\left\{\begin{array}{c} \alpha_{i,j^{\prime }}\cdot m_{i}\left(A\right),\\ 1-\sum_{B\subset \theta }\alpha_{i,j^{\prime }}\cdot m_{i}\left(B\right) \end{array}\right.\begin{array}{c} A\subset \theta \\ B\subset \theta ,A=\theta \end{array}$$

where $\alpha_{i,j^{\prime }}$ is the combination of sufficiency index *μ* and importance index *v*, which is defined as $\alpha_{i,j^{\prime }}=v_{i,j^{\prime }}\cdot \mu_{i}$.

After modification, if the BBAs are still in conflict with each other, Fan and Zuo proposed to use the non-conflict factor to modify Dempster’s combination rule. For more detailed information, please refer to [43].

Though Fan and Zuo’s method can handle the conflict problem and perform data fusion effectively, it has some limitations. First, it only considers the static reliability of sensors, such as evidence sufficiency and evidence importance, and ignores the dynamic reliability, which is reflected in the real-time detection process; it is not reasonable in practice. Besides, Fan and Zuo introduced a process of judging the conflict degree between pieces of evidence, according to the degree of different combination rules adopted, which makes it much more complex to make a decision.

## 3. The Proposed Method

### 3.1. Static Reliability

The sensor reliability is of great value in comprehending and quantifying the sensor performance. Whether the fusion result is reasonable is closely associated with the static reliability of sensors, such as accuracy, work efficiency and experts with different knowledge. The sensor static reliability can be affected by technical factors and noise, such as principle, material, manufacturing craft, and so on. It can be evaluated by comparing the sensor outputs with the actual values in long-term practical applications. In this paper, we adopt evidence sufficiency and evidence importance in Fan and Zuo’s method [43] to measure the static reliability of sensors. The static reliability index is denoted as $w^{s}$, where superscript *s* means “static reliability”. $w^{s}$ combines the sufficiency index and the importance index, which is defined below:

(17) $$w^{s}=\mu_{i}\times \nu_{i,j^{\prime }}=\alpha_{i,j^{\prime }}$$

If an evidence has a high sufficiency level and a high level of importance, it will be assigned a high weight, so that it can have a great effect on the final data fusion result and the decision making.

### 3.2. Dynamic Reliability

The sensor reliability is also related to the target and surrounding properties, such as environment noises, the presence of unknown targets and the deception behaviors of observed targets [44]. Due to different sources, different sensors have different adaptationsto the environment. Hence, it is also important to take the dynamic reliability of sensors in the combination process into consideration. The dynamic reliability is generally evaluated by measuring the consensus among a group of sensors. As for the same input, the sensors may have different reports. If a sensor report reaches a good consensus with those of other sensors, it has good adaptive performance to the environment, which means it is stable and reliable in detection. From this point of view, the weight assigned to this type of sensor is supposed to be great to guarantee that it can play a more important part in the final combination result and decision making. On the contrary, if a sensor has poor adaptability to the environment, the weight assigned to this kind of sensors should be small, so that it has little influence on the final result.

Evidence distance is an efficient tool to measure the dissimilarity between every two pieces of evidence [50]. Additionally, it can be adopted to reflect the consensus among the sensors, which can be used to evaluate the dynamic reliability. If evidence has a large distance from others, it is poorly supported by other evidence, namely it has great conflict with the others, which means it has a lower level of credibility and has little consensus with the others. To reduce the influence of such evidence on the final decision, it will be assigned small weights.

In this paper, one contribution is that not only evidence distance, but also Deng entropy are introduced to measure the information volume of the evidence [51]. Suppose that there is evidence containing a great volume of information; it is supposed to have a little conflict with others; in other words, it has great consensus with other evidence and is well supported by others. As for this kind of evidence, it will be distributed with a big weight to have a great effect on the final decision.

The dynamic reliability combining both evidence distance and Deng entropy denoted $w^{d}$ is defined as:

(18) $$w^{d}=Crd_{i}\times Ed_{i}$$

where the superscript *d* of $w^{d}$ represents “dynamic reliability”. $Crd_{i}$ is calculated by Equation (12). $Ed_{i}$ is obtained after normalization of Equation (14). The process is given below:

(19) $$Ed\left(i\right)=\frac{Ed(i)}{maxEd(i)}$$

If evidence has a high dynamic reliability that is equal to one, this means this piece of evidence is completely reliable. Besides, this piece of evidence has not only the highest credibility, but also the maximum information volume. In addition, this evidence will play a significant part in the final result. On the contrary, if evidence has a very low dynamic reliability, which equals zero, this means this evidence highly conflicts with others, and it may distribute the whole BBA to a single element. It is obvious that this type of evidence will not participate in decision making. The dynamic reliability considering both Deng entropy [51] and evidence distance [50] can reflect the adaptability and the dynamic reliability of the sensors effectually.

### 3.3. Comprehensive Reliability of Sensor

Based on all of the above, this paper proposes a new comprehensive method to model the sensor reliability. The new model combining the static reliability and dynamic reliability is more reasonable. It is defined below:

(20) $$w=w^{s}\times w^{d}$$

where $w^{s}$ is given in Equation (17) and $w^{d}$ is obtained by Equation (18). According to the sensor reliability, the weight of each piece of evidence can be obtained. It is obvious that the more reliable the sensor is, the greater effect it has on the fusion result, which is great help for making the right decision.

Suppose there are *n* pieces of evidence, the final weights on the basis of Equation (20) after normalization is given below:

(21) $$w\left(i\right)=\frac{w(i)}{\sum_{i=1}^{n}w\left(i\right)}$$

**Figure 1:**
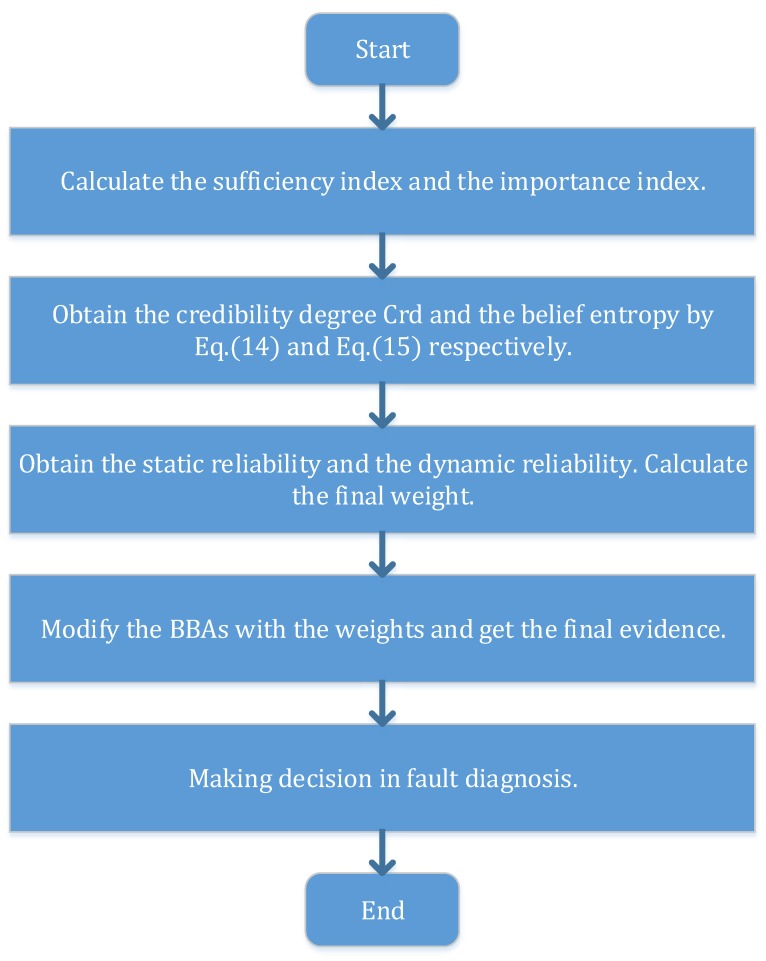
The flowchart of the new method.

Use the value to make weighted averaging according to Equation (13), and the weighted evidence can be obtained. Combine the weighted evidence n−1 times, and the fusion result can be obtained to make the final decision. To illustrate the process more clearly, Figure 1 shows the flowchart of the new method specifically.

**Figure 2:**
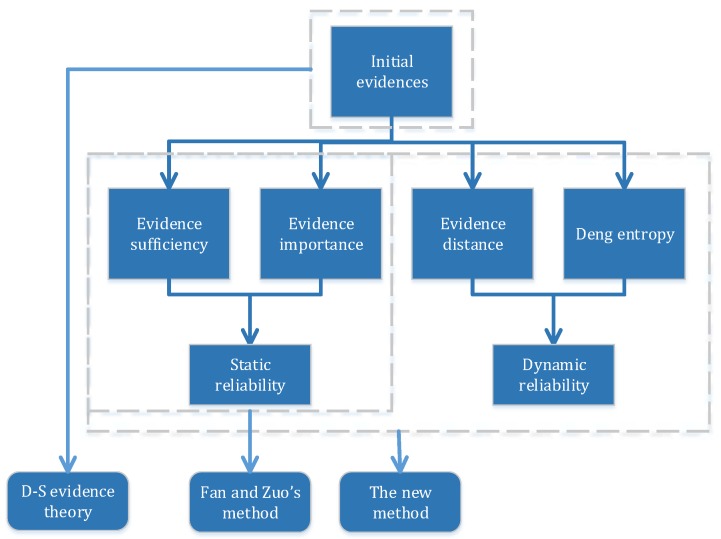
The relationship between Fan and Zuo’s method and the new method.

In Fan and Zuo’s method [43], the evidence sufficiency and evidence importance both belong to static reliability, which neglects the significance of dynamic reliability. In contrast, the new method is more reasonable and considerate. The relationship between Fan and Zuo’s method and the new method is illustrated in Figure 2.

D-S evidence theory simply combines the initial evidence to make a decision [43]. While in the other two methods, the reliability of sensors is also taken into consideration. Fan and Zuo’s method [43] only requires the input of evidence sufficiency and evidence importance [43]. However, it is not easy to obtain these parameters in practice. In comparison, the new method is more reasonable and has greater consideration, which makes a great contribution to improve the accuracy of decision making.

## 4. Application

The example from paper [43] is given to demonstrate the effectiveness of the new method.

Example 5: Assume a machine has three gears $G_{1}$, $G_{2}$ and $G_{3}$, and the failure modes $F_{1}$, $F_{2}$, $F_{3}$ represent that there are faults in $G_{1}$, $G_{2}$, and $G_{3}$, respectively. The fault hypothesis set is $\theta =\left\{F_{1},F_{2},F_{3}\right\}$. Suppose there are three types of sensors named $S_{1}$, $S_{2}$ and $S_{3}$, respectively. Additionally, the evidence derived from different sensors is denoted by $E=\left\{E_{1},E_{2},E_{3}\right\}$. The BBAs based on these pieces of evidence are given in Table 1.

**Table 1:** Basic belief assignments (BBAs) for the example.

	$\left\{F_{1}\right\}$	$\left\{F_{2}\right\}$	$\left\{F_{2},F_{3}\right\}$	*θ*
$E_{1}:m_{1}\left(\cdot \right)$	0.6	0.1	0.1	0.2
$E_{2}:m_{2}\left(\cdot \right)$	0.05	0.8	0.05	0.1
$E_{3}:m_{3}\left(\cdot \right)$	0.7	0.1	0.1	0.1

The conflict factors between each pair of evidence are $k_{1,2}=0.52$, $k_{1,3}=0.26$, $k_{2,3}=0.605$. It is obvious that the second piece of evidence conflicts highly with the others. Assume the sufficiency indexes of the three pieces of evidence are 1, 0.6, 1, respectively. Additionally, the importance indexes are 1, 0.34, 1.

According to Equation (12), the credibility degree $Crd_{i}$ of these three pieces of evidence can be calculated based on the initial BBAs.

$$\begin{array}{c} Crd_{1}=1.0000\\ Crd_{2}=0.5523\\ Crd_{3}=0.9660 \end{array}$$

Adopt Equation (14) to calculate the Deng entropy, which is given below:

$$\begin{array}{c} E_{d1}=2.2909\\ E_{d2}=1.3819\\ E_{d3}=1.7960 \end{array}$$

Additionally, the results after normalization of the Deng entropy according to Equation (19) are as follows:

$$\begin{array}{c} \varphi_{1}=1.0000\\ \varphi_{2}=0.6032\\ \varphi_{3}=0.7840 \end{array}$$

Then, the static reliability $w^{s}$ and the dynamic reliability $w^{d}$ can be obtained based on Equations (17) and (18), respectively. On basis of all of the above, the final weights according to Equation (20) are given below:

$$\begin{array}{c} w_{1}=1\times 1\times 1\times 1=1\\ w_{2}=0.6\times 0.34\times 0.5523\times 0.6032=0.0680\\ w_{3}=1\times 1\times 0.9660\times 0.7840=0.7573 \end{array}$$

The final weights after normalization are shown as follows:

$$\begin{array}{c} w_{1}=0.5479\\ w_{2}=0.0372\\ w_{3}=0.4149 \end{array}$$

Use the weights to modify the BBAs, and the results are given below:

$m\left(\left\{F_{1}\right\}\right)=0.6210,m\left(\left\{F_{2}\right\}\right)=0.1261,m\left(\left\{F_{2},F_{3}\right\}\right)=0.0981,m\left(\Theta \right)=0.1548.$

After the combination by Equation (3), the final BBAs are:

$$\begin{array}{c} m(\left\{F_{1}\right\})=0.8948\\ m(\left\{F_{2}\right\})=0.0739\\ m(\left\{F_{2},F_{3}\right\})=0.0241\\ m(\Theta )=0.0072 \end{array}$$

Table 2 shows the results obtained by different methods.

**Table 2:** Comparison between the proposed method and other methods. D-S, Dempster–Shafer.

	$\left\{F_{1}\right\}$	$\left\{F_{2}\right\}$	$\left\{F_{2},F_{3}\right\}$	*θ*
D-S evidence theory	0.4519	0.5048	0.0336	0.0096
Fan and Zuo’s method [43]	0.8119	0.1096	0.0526	0.0259
The proposed method	0.8948	0.0739	0.0241	0.0072

According to the proposed method, the fault $F_{1}$ has a belief degree of 89.48%, while the fault $F_{2}$ only has a belief degree of 7.39%. It is clear that $m\left\{F_{1}\right\}>m\left\{F_{2}\right\}$. Therefore, we can find that the fault is $F_{1}$, which means that Gear 1 has a fault.

In D-S evidence theory, the BBA of fault $F_{1}$ is 0.4519, while that of $F_{2}$ is 0.5048. Due to the conflict evidence $E_{2}$, D-S evidence theory comes to the wrong result that $m\left\{F_{2}\right\}>m\left\{F_{1}\right\}$, which may lead to the wrong decision; while the other two methods deal with the conflict evidence $E_{2}$, so that they can both reach the right result.

In Fan and Zuo’s method, the belief degree of $F_{1}$ is 81.19%, while the new method has a higher belief degree of 89.48%. The main reason is that the proposed method takes into consideration not only the static reliability represented by evidence sufficiency and evidence importance, but also the dynamic reliability measured by evidence distance and entropy, which decreases the conflict evidence $F_{2}$’s influence on the final result enormously.

The new method improves the accuracy of fault diagnosis from 81.19% to 89.48%, which illustrates the efficiency of the new method in conflict management and fault diagnosis.

## 5. Conclusions

How to efficiently model sensor reliability greatly affects the performance of the sensor fusion system. To address this issue, a new sensor reliability model combining both dynamic reliability and static reliability is presented in this paper. The dynamic property of the sensor reliability is determined by the distance function of the sensor report and the information volume of each sensor report. A new discounting coefficient is proposed to improve the classical Dempster combination rule. An application in fault diagnosis is illustrated to show the efficiency of our proposed method. It seems that our proposed method is more efficient for handling highly conflicting evidence. In addition, from the result obtained in this paper, the new method can identify the fault correctly and improve the accuracy of fault diagnosis from 81.19% to 89.48%.

The proposed method has two aspects of merit. From the aspect of the math model of sensor data fusion, the proposed work takes into consideration not only evidence distance, but also the information volume of the sensor itself, which contributes to the dynamic property of sensor reliability more reasonably. From the aspect of a real application in fault diagnosis, the proposed method can effectually handle conflict management. The application results show that the accuracy in fault diagnosis is improved from 81.19% to 89.48%. Besides, it can be easily extended to other multi-sensor systems, which makes it useful to practical engineering.
